# Genetic Diversity and Population Structure Reveal Post-Introduction Differentiation in *Heracleum sosnowskyi*

**DOI:** 10.3390/genes17050502

**Published:** 2026-04-24

**Authors:** Anna Rysiak, Sylwia Sowa, Mariusz Kulik, Aneta Koroluk, Joanna Lech, Piotr Kacorzyk, Agnieszka Klarzyńska, Teresa Wyłupek, Edyta Paczos-Grzęda

**Affiliations:** 1 Department of Botany, Mycology and Ecology, Maria Curie-Skłodowska University, Akademicka 19, 20-033 Lublin, Poland; anna.rysiak@mail.umcs.pl; 2 Institute of Plant Genetics, Breeding and Biotechnology, University of Life Sciences in Lublin, Akademicka 15, 20-950 Lublin, Poland; sylwia.sowa@up.edu.pl (S.S.); aneta.koroluk@up.edu.pl (A.K.); joanna.toporowska@up.edu.pl (J.L.); edyta.paczos@up.edu.pl (E.P.-G.); 3 Department of Grassland and Landscape Planning, University of Life Sciences in Lublin, Akademicka 15, 20-950 Lublin, Poland; teresa.wylupek@up.edu.pl; 4 Department of Agroecology and Plant Production, University of Agriculture in Krakow, Al. Mickiewicza 21, 31-120 Krakow, Poland; piotr.kacorzyk@urk.edu.pl; 5 Department of Grassland and Natural Landscape Sciences, Poznan University of Life Sciences, Dojazd 11, 60-632 Poznan, Poland; agnieszka.klarzynska@up.poznan.pl

**Keywords:** gene flow, genetic differentiation, invasion biology, adaptive processes, geographic variation

## Abstract

Background/Objectives: Sosnowsky’s hogweed Heracleum sosnowskyi, which originated in the Greater Caucasus region and spread rapidly across Central and Eastern Europe after being introduced as cattle fodder in the 1950s, is an example of an extremely dangerous invasive species listed by the European Union. This study aimed to estimate the genetic diversity of 6 native populations of Sosnowsky’s hogweed from the Caucasus region of Russia and Georgia, as well as 15 invasive populations from Lithuania and Poland, and to assess the adaptability of hogweed in new environments. Methods: Genetic analyses of plant material were conducted, including DNA extraction, ISSR genotyping, PCR product separation, and subsequent molecular data mining and analysis. Results: A pairwise Mantel test revealed a positive correlation between geographical distance and the genetic diversity of the hogweed populations. The presence of three distinct allele pools was confirmed in the populations under study, with genotypes from Poland dominated by the first allele pool, which had the largest number of polymorphic and private loci. Analysis of molecular variance by origin showed that 99% of the variation was within the analysed hogweed populations, with only 1% being between them. Native populations from Russia were genetically distinct from those in Poland and Lithuania. Some of the Georgian population shows genetic similarities to Russians, while the rest shows similarities to the secondary invasive Lithuanians. Conclusions: Introduced populations of H. sosnowskyi are characterised by considerable genetic variation, likely resulting from multiple introductions and subsequent evolutionary processes, which may facilitate local adaptation and invasiveness, although overall large-scale genetic differentiation remains low.

## 1. Introduction

For invaders to establish themselves in a recipient environment, they must first pass through the “ecological filter” of that environment, which is composed of two major components, biotic and abiotic [1,2]. Invasive species frequently encounter novel ecological contexts [3,4,5], both biotic (e.g., pathogens) and abiotic (such as high temperature exposure) [6].

Fodder shortages in Europe after the Second World War prompted researchers to look for plants that were easy to grow, produced large amounts of biomass, and could easily adapt to different habitat conditions, especially in cold climates. One plant that met these criteria was Sosnovski’s hogweed [7]. Intentional introduction has been the main source of the species in the past. Seeds of *Heracleum sosnowskyi* used in the first experiments, carried out at the Polar–Alpine Botanical Garden-Institute (PABGI), Kirovsk, North Russia, in 1946–1947 and at the Komarov Botanical Institute, St. Petersburg, in 1953, were obtained from Kabardino-Balkaria (North Caucasus, Russia). Later reports have mentioned seeds from other sources, e.g., Dagestan, Eastern Caucasus, Russia [8,9,10]. Breeding programmes spread to many other places and *H. sosnowskyi* was cultivated for biomass and silage production in the former USSR (including locations such as Belarus, Estonia, Latvia, Lithuania, Ukraine). The plant appeared in Poland in the late 1950s, having been introduced from central Russia as a potential fodder, medicinal, and ornamental plant [11,12,13]. Cultivation of *H. sosnowskyi* for forage has been abandoned in most places, for two main reasons: the anise-scented plants affected the flavour of meat and milk from animals that eat them, and they posed a health risk to humans and livestock [7,14].

Since the 1990s, the plant was reported to have escaped from cultivation and naturalised. It is now widespread in the Baltic region and much of European Russia [7,15]. In Europe, *H. sosnowskyi* is mainly distributed in the eastern parts, reflecting the history of planting in the former USSR. The most infested areas are Estonia, Latvia, Lithuania, Ukraine, and Poland. Secondary habitats are mainly roadsides, disturbed areas, outskirts of agricultural fields, abandoned yards and gardens and semi-natural habitats like: bushes, grasslands, river valleys and forest glades [16,17,18]. *H. sosnowskyi* is naturally dispersed by water and wind [17,19]. Seeds can also be transported attached to animal fur—of sheep and cattle—as reported by [7], by soil movement during construction and excavation, by vehicles or by air currents of roads or railways, and by the movement of agricultural and forestry tractors carrying seeds on radiators and roofs [20].

Genetic diversity, and thus the adaptive potential of invasive populations, is largely based on three factors: patterns of genetic diversity in the species’ native range, the number and location of introductions and the number of founding individuals per introduction [21]. After the introduction of an invasive species into a target area, the level of genetic diversity within populations is often reduced in the first stage of expansion [22,23]. However, after a lag, the genetic diversity can be restored [24,25,26]. Exposure to novel biotic or abiotic conditions can induce stress in some plants, which has been shown to affect genome stability in some instances and introduce genetic diversity [27,28].

Therefore, by understanding the level of genetic diversity, it should be possible to predict the further spread of the species. In the present study, we aim to characterise the genetic diversity and structure of *H. sosnowskyi* populations. To assess DNA-level genetic polymorphism, we used Inter Simple Sequence Repeat (ISSR) markers, a reproducible and cost-effective technique suitable for detecting genome-wide variation without prior sequence information [23,29]. In particular, we intended to assess genetic diversity and differentiation within and between populations of native and invasive origin. The invasion of Sosnowsky’s hogweed has been linked to its deliberate introduction into agricultural areas and its cultivation as a fodder crop. The indirect aim of our work is to learn more about the adaptation mechanisms of the studied species and to use this knowledge for the conservation of biodiversity and ecological restoration of areas occupied by *H. sosnowskyi* and ultimately to prevent its spread to agricultural areas.

## 2. Materials and Methods

### 2.1. Study Area

**Figure 1:**
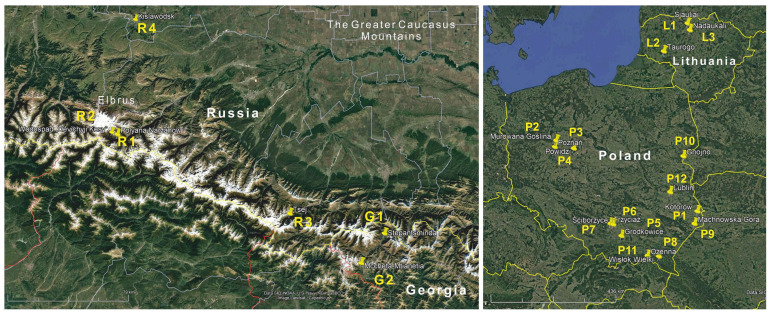
Geographical distribution of native populations of *H. sosnowskyi* Manden located in Georgia: Mccheta-Mtianetia (G2), Stepantsminda (G1) and Russia: Kislawodsk (R4), Polyana_Narzanov (R2), Tsey (R3), Vodopad “Devichji Kosy” (R1), and secondary, invasive located in Poland: Gnojno (P10), Grodkowice (P5), Kotorów (P1), Lublin (P12), Machowska Góra (P9), Murowana Goślina (P2), Ożenna (P8), Powidz (P4), Poznań (P3), Ściborzyce (P7), Trzyciąż (P6), Wisłok Wielki (P11), and in Lithuania: Nadaukali (L3), Siauliai (L1), Taurage (L2).

Twenty-one populations of *H. sosnowskyi* were studied: six native populations were from the region of origin of the study species, Georgia [2] and Russia [4], while the remaining fifteen populations, classified as invasive, were from Lithuania [3] and Poland [12] (Figure 1). The populations at each site were spatially isolated from each other and interbreeding was not possible. Sampling sites were selected to represent both native (Georgia, Russia) and invasive (Lithuania, Poland) populations, and at each site samples were collected from multiple scattered locations to ensure representativeness. The study area covered different ecosystems: grassland (including pastures and meadows), wasteland (including ruderal areas and abandoned grassland) and forest (including woodland). The distribution of *H. sosnowskyi* varied from very small patches (0.01 ha) to very large patches (1.2 ha). The study areas also varied in altitude (a.s.l.) and exposure. Plant samples were collected from 18 June to 26 August 2023 (Table 1).

### 2.2. Study Species and Plant Material

*H. sosnowskyi* was described in 1944 [30] from the Meskhetia district of Georgia. It is native to the Caucasus Mountains, where it grows in medium to high altitude meadows [10]. Countries in this region that have *H. sosnowskyi* in their native flora are Georgia, Russia, Armenia, Azerbaijan and Turkey. In natural habitats, *H. sosnowskyi* forms large stands, e.g., in meadows, river valleys and forest margins, as well as on the floodplains of rivers and lakes.

The plant’s large size, high fecundity, early germination and vigorous growth make it a very successful invader, able to outcompete the local flora [30,31,32]. One of the main reasons for its harmfulness is its phototoxic effect, which causes skin burns from direct contact with the plant in the presence of sunlight. The substances responsible for this effect are called furanocoumarins and are found in the aerial parts of the plant, particularly the leaves and stems [6,33,34].

Plant samples were collected from specimens of *H. sosnowskyi* belonging to native and invasive populations. From each site, 5 leaves were collected from different plant specimens scattered throughout the study area at least 15 m apart (Figure 1).

The leaves were dried in silica gel until completely dry. During the field work, the area, location and habitat, development stage and plant height were determined for each population (Table 1).

### 2.3. DNA Extraction

Total genomic DNA was extracted from lyophilised leaf tissue using the GeneMATRIX Plant Fungi DNA Purification Kit (EURx) and purified using the Anti-Inhibitor Kit (AA Biotechnology, Gdansk, Poland). DNA quality and integrity were assessed by electrophoresis on 1% agarose gel. DNA concentration was assessed by NanoDrop2000 spectrophotometry and normalised to 10 ng/μL.

### 2.4. ISSR Genotyping

PCR reactions were performed according to the Inter Simple Sequence Repeat (ISSR) method [35] with modifications. A 12 µL reaction mixture contained template DNA (20 ng), 1 × PCR Master Mix buffer (0.05 U/μL Taq DNA polymerase, 4 mM MgCl2, 0.4 mM of each dNTP; Thermo Fisher Scientific) and 0.6 nM primer (Table 2). The following temperature profile was used: 95 °C for 7 min (predenaturation); 3 cycles of 95 °C for 30 s, 54 °C for 45 s and 72 °C for 2 min; 3 cycles of 95 °C for 30 s, 53 °C for 45 s and 72 °C for 2 min; 32 cycles of 95 °C for 30 s, 52 °C for 45 s and 72 °C for 2 min; and a final extension of 72 °C for 7 min.

### 2.5. PCR Products Separation

Amplification products were visualised on a 1.5% agarose gel containing 5 μg/mL EtBr in 1/TBE buffer (90 mM Tris-borate, 2 mM EDTA, pH 8.0) Gene RulerTM 100 bp Plus DNA Ladder was used to determine the molecular weight of the products. DNA fragments were photographed under a UV transilluminator.

### 2.6. Molecular Data Mining and Analysis

ISSR fragments were transformed into a 0/1 matrix by coding the presence of the fragment as 1 and its absence as 0. Marker information was calculated for each primer by counting NPF—number of polymorphic fragments and PIC—polymorphism information content. PIC was calculated as the mean of all PIC values for all amplified fragments according to the following formula:

$$PIC=2*f_{i}*(1-f_{i})$$

where *f_i_* is the frequency of the amplified allele (band present) and (1 − *f_i_*) is the frequency of the null allele (band absent) [36,37].

Unbiased expected heterozygosity (He), percentage of polymorphic products and private bands were estimated using GenAlex 6.502 [38]. Genetic distance was calculated using the Nei formula [39].

Analysis of molecular variance (AMOVA) within and between groups of individuals divided on the basis of collection site was determined by 999 permutations. Relationships between all genotypes examined were assessed using the Unweighted Pair Group Method with Arithmetic Mean (UPGMA) cluster analysis on [40] similarity scores with 1000 bootstraps and principal coordinate analysis (PCoA) in PAST 3.25 [41]. Based on the geographic coordinates of the collection sites, the matrix of geographic distance was generated. The correlation between geographic and genetic distances between samples was tested by non-parametric Mantel tests with 999 permutations [42] in GenAlex 6.502 (H0 = no correlation between matrices, Rxy = 0).

The population structure was estimated using a Bayesian approach implemented in the STRUCTURE 2.3.4 software [43] with the admixture model and correlated allele frequencies between populations. The number of clusters (K) was set from 2 to 21, with ten independent runs for each k (10,000 burn-ins and 100,000 Markov chain Monte Carlo replications). StructureSelector [44], which integrates the Clumpak programme [45], was used to select and visualise the optimal number of clusters.

## 3. Results

### 3.1. ISSR Amplification

**Figure 2:**
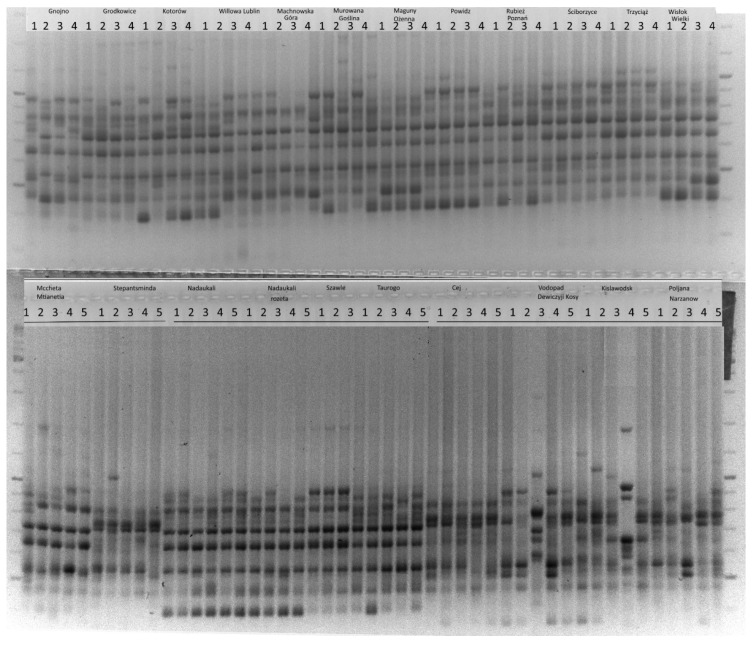
ISSR amplification profile of *H. sosnowskyi* populations originating from the native range (Georgia, n = 2; Russia, n = 4) and invasive populations from Lithuania (n = 3) and Poland (n = 12). Amplification was performed using primer SR50. GeneRuler 100 bp Plus DNA Ladder was used as a molecular size standard on both sides of the gel.

In the study, 63 ISSR primers were used for preliminary DNA profiling. Only 17 ISSR primers amplified polymorphic products and were subjected to further analysis. A total of 244 polymorphic amplification products were obtained (Table 2, Figure 2). The amplified fragments ranged from 220 to 1900 bp depending on the primer used. On average, a single ISSR amplified 14 polymorphic DNA fragments (range 7–19). ISSR primers SR45, SR63 and SR64 were the most efficient, each producing 19 polymorphic bands. The least efficient was SR35, which amplified seven polymorphic loci. To identify the most informative primers, PIC parameters were calculated for each primer. The PIC values ranged from 0.293 (SR17) to 0.433 (SR54) The average PIC for the ISSR method was 0.347.

### 3.2. Genetic Diversity

**Figure 3:**
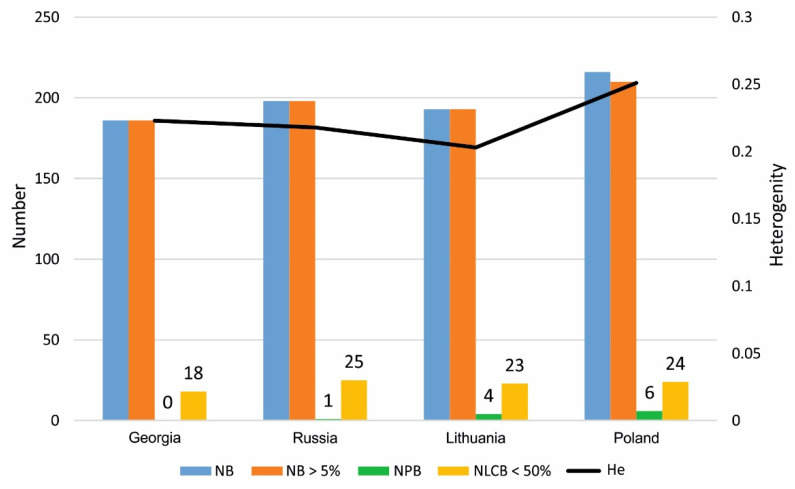
Band patterns across *H. sosnowskyi* populations. No. of different bands (NB); no. of different bands with a frequency ≥ 5% (NB > 5%); no. private bands (NPB) = no. of bands unique to a single population; no. locally common bands (NLCB) (≤50%) = no. of (Freq. ≥ 5%) found in 50% or fewer group (NLCB < 50%); unbiased expected heterozygosity (He) = (2N/(2N − 1) (He); no. locally common bands (≤25%) = no. of locally common bands (Freq. ≥ 5%) found in 25% or fewer group.

ISSR amplification results were used to determine the level of genetic diversity within the 96 *H. sosnowskyi* genotypes collected in invasive populations—Poland, Lithuania and native populations—Georgia, Russia (Figure 2). The average percentage of polymorphic loci among genotypes collected in different countries was 70.08 ± 5.8%. The highest value was observed for genotypes collected in Poland (77.05%) and the lowest for genotypes collected in Lithuania (62.70%). The highest number of private bands (unique to the group) was observed for plants collected in Poland (six bands) The number of private bands for *H. sosnowskyi* collected in Lithuania and Russia was four and one, respectively. No private bands were observed for genotypes collected in Georgia. The mean unbiased expected heterozygosity (uHe) of the distinguished groups was 0.231 (Table 3, Figure 3).

The AMOVA study performed to determine the genetic diversity between and within *H. sosnowskyi* genotypes collected in four different countries showed that 99% of the variation was within the analysed groups and 1% between groups. The pairwise genetic distance, expressed in PhiPT, between the studied populations was PhiPT = 0.009, *p* = 0.09 (Table 4a). When the genotypes were grouped into native population (from Russia and Georgia) and invasive population (from Poland and Lithuania), 98% of the variation was found within groups and 2% between groups (PhiPT = 0.018, *p* = 0.001) (Table 4b).

Comparisons of significant genetic differences between populations clarified the patterns of variation. Genetic differentiation was found between the native population from Russia and the invasive population from Poland (PhiPT = 0.024, *p* = 0.001) as well as the invasive population from Lithuania (PhiPT = 0.017, *p* = 0.046; Table 5a). There was also a significant difference between the native population formed by genotypes from Russia and Georgia and the invasive population formed by plants from Poland and Lithuania (PhiPT = 0.018, *p* = 0.002). The pairwise Mantel test showed a statistically significant positive correlation between geographic distance and genetic variation (r = 0.716, *p* = 0.01).

**Figure 4:**
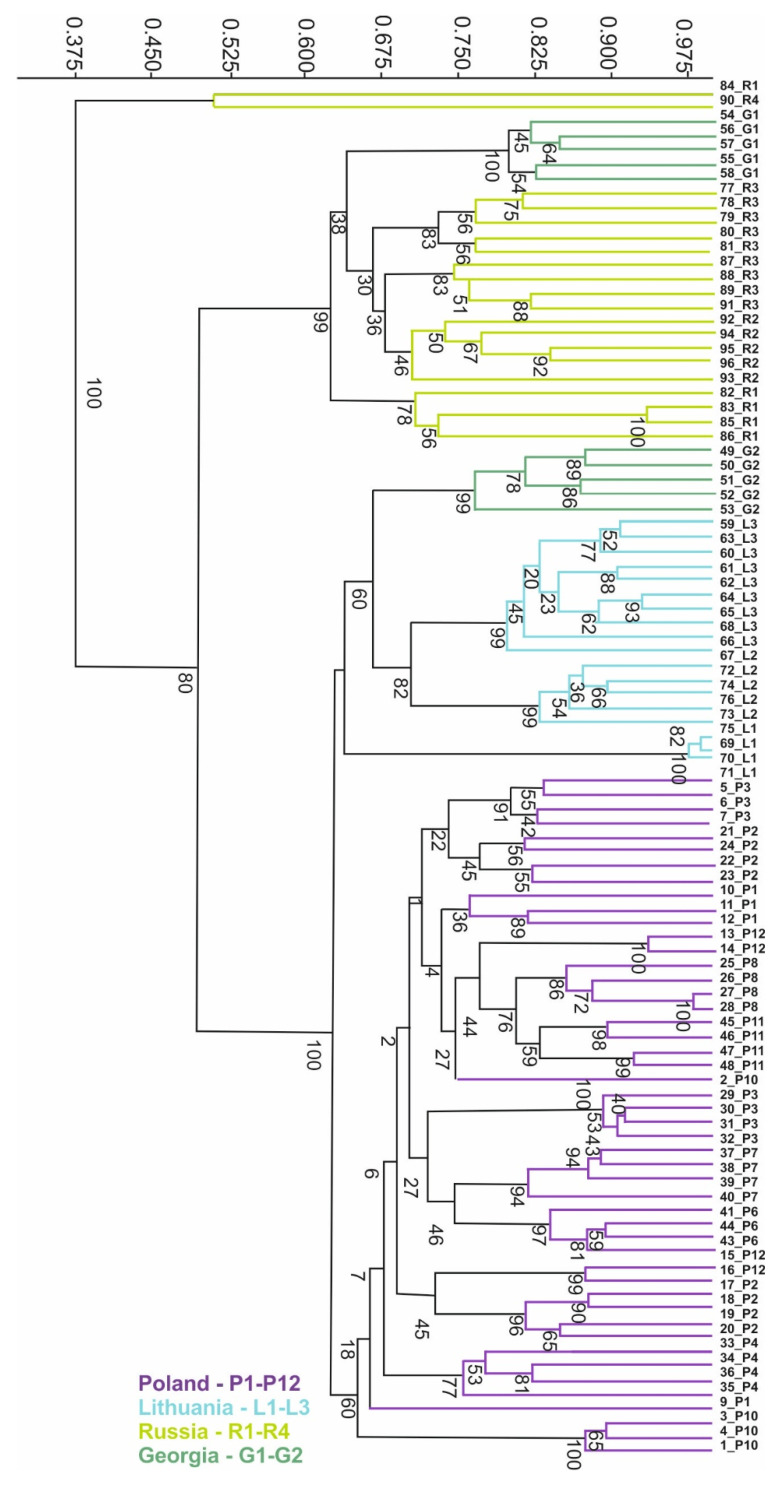
Dice’s similarity UPGMA dendrogram of *H. sosnowskyi* genotypes collected in Poland (P1–P12; 1–48), Georgia (G1–G2; 49–58), Lithuania (L1–L3; 59–76) and Russia (R1–R4; 84–96), based on ISSR markers. Bootstrap value indicated on clods.

The mean value of Dice’s genetic similarity coefficient between the analysed genotypes was 0.601 and ranged from 0.275 between objects from Russia (90_R3) and Poland (sample 9_P1) to 0.987 between objects from Lithuania (samples 69_L2 and 70_L1). Four clusters were distinguished on the UPGMA dendrogram (Figure 4). The first cluster contained all genotypes from Poland. The second cluster consisted of genotypes from Lithuania and four objects from Georgia, collected in Mccheta-Mtianetia (samples 49 and 53_G2). The third cluster included objects from Russia and the remaining genotypes from Georgia collected in Stepantsminda (54 and 58_G1). The fourth and most distant cluster included two genotypes collected in Russia (samples 84_R2 and 90_R4).

**Figure 5:**
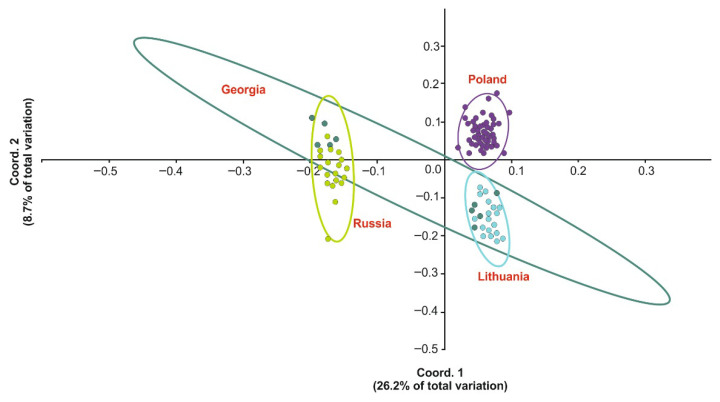
Principal coordinates (PCoA) 1 vs. 2 plotted for 96 *H. sosnowskyi* genotypes collected in Poland [48 genotypes], Georgia [10 genotypes], Lithuania [18 genotypes] and Russia [20 genotypes], based on Dice similarity index. Each point represents a separate population.

Principal coordinate analysis based on both ISSR markers (Figure 5) gave congruent results, confirming the cluster analyses. The first two coordinates explained 34.9% of the variance. The PCoA analysis clearly separated the genotypes of the invasive population in Poland from the others. Polish populations are genotypically similar to Lithuanian populations. The Russian and Georgian populations, which occur in the natural range of the studied species, are genotypically similar. There is also a clear similarity between selected Georgian populations and those recorded in Lithuania.

### 3.3. Genetic Structure

**Figure 6:**
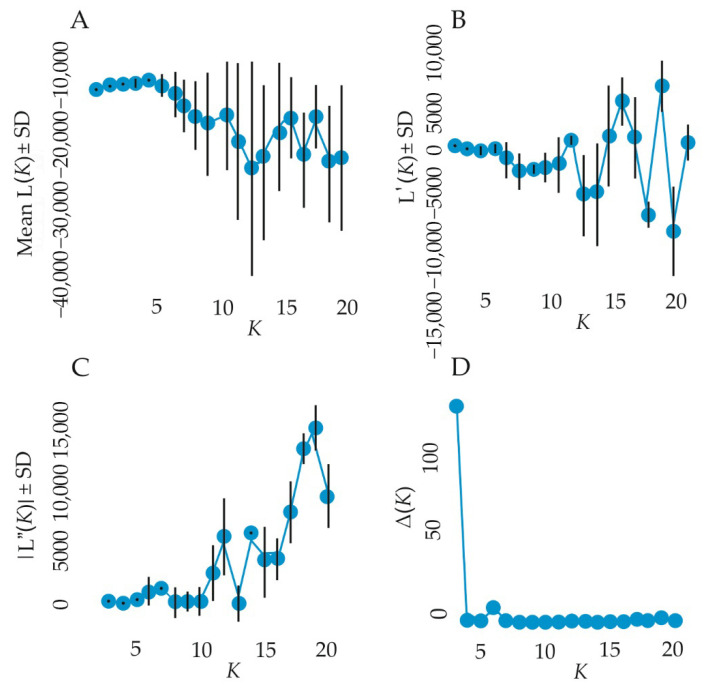
Estimated number of clusters of *H. sosnowskyi* genotypes collected in study populations obtained for K values from 2 to 21 using ISSR data based on ΔK. (**A**) Mean log-likelihood of data [Ln P(D)] ± SD across K. (**B**) First-order difference of Ln P(D), L′(K) ± SD. (**C**) Second-order difference of Ln P(D), |L″(K)| ± SD. (**D**) ΔK statistic identifying optimal K.

The genetic structure of *H. sosnowskyi* genotypes was estimated by model-based Bayesian clustering using STRUCTURE software. The ΔK statistical test showed that K = 3 was optimal in this analysis, supporting the presence of three distinct allele pools (Figure 6A–D).

**Figure 7:**
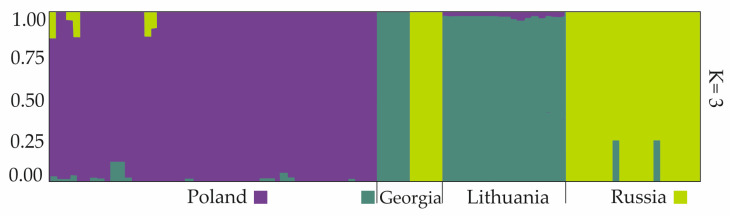
The graphic representation of Bayesian analysis of *H. sosnowskyi* genotype structure (Poland 1–48, Georgia 49–58, Lithuania 59–76 and Russia 84–96) for K = 3. Different colours clustersrepresent diverse genetic backgrounds/lines.

Genotypes from Poland were dominated by the first allele pool. The second allele pool was predominant in genotypes from Lithuania. Plants from Russia were mostly the third pool, with (samples 84_R2 and 90_R4) being mixtures of the second and third allele pools. Within the genotypes from Georgia, plants collected in Mccheta-Mtianetia (samples 49 and 53_G2) were dominated by pool 2 and plants collected in Stepantsminda (samples 54 and 58_G1) were dominated by pool 3 (Figure 7).

The graphical representation of the structure is consistent with the UPGMA and PCoA analysis (Figure 5).

## 4. Discussion

### 4.1. Genetic Variation

The analysis was based on ISSR marker polymorphisms. Values of marker polymorphism indexes confirmed that the applied method of polymorphism evaluation was appropriate, and the ISSR primers used were characterised by similar informativeness. The 244 polymorphic products obtained during analysis were then used to assess the genetic diversity and population structure of the studied materials. The largest number of polymorphic products was found among populations originating from Poland. Also, in these populations, the largest number of private bands was observed, which most likely indicate changes in the genome as a result of adaptation. Some authors [27,28] have previously demonstrated the occurrence of such changes under the pressure of new environmental conditions. A relatively small number of polymorphic fragments was found in native populations, which may indicate a relatively poor genetic pool of this species in its place of origin. Numerous studies have reported higher genetic variation in the native compared to the introduced populations, reviewed in [46], while few studies have also reported similar and lower levels of genetic variation in the native relative to the introduced populations [25,47,48,49]. Our working hypothesis assumed that the introduced populations of *H. sosnowskyi* occurring in Lithuania and Poland would possess a low level of genetic variation and considerable differentiation among populations. However, the amount of genetic variation measured as expected heterozygosity was lower in the Lithuanian population (UHe = 0.206) and slightly higher in the Polish population compared (UHe = 0.254) to the native population. In previous genetic studies on *H. sosnowskyi* and Heracleum genus, both high [10,23,50,51] and low genetic diversity [52] have been detected in the invaded regions.

Our analyses showed that most of the allelic variation (77%) was found in the introduced, invasive range (populations in Poland). They are comparable to the results obtained for the naturally occurring population of Russia. A second population in the secondary range of hogweed, found in Lithuania, has allelic variability comparable to that shown for Georgia: 62.7% and 66.39%, respectively. Different results were obtained for *Heracleum persicum* [53], where most of the allelic variation (97%) was found in the native range, while less than half of the allelic variation found in the native range was retained in populations introduced to Europe. Furthermore, the total number of private alleles in the invasive range (90.86%) was much higher than in the native Caucasian range (9,09%). The opposite results were obtained for *H. persicum*, where the total number of alleles unique to the native range (54%) was much higher than in the European range (3%), demonstrating the strong founder effect, especially in Norwegian populations [53]. The highest number of private alleles occurring in the Polish population (54.5%) proved that they are more distant from native populations than populations from Lithuania. Here we can speculate about the rapid evolution and adaptation to new conditions of the Polish Heracleum populations. Our results are consistent with those of other ISSR studies of invasive plant species, which often exhibit significant genetic variability within introduced populations. Differences in diversity patterns observed in studies by other authors may reflect different introduction pathways, invasion genesis, or species-specific demographic processes.

Analysis of molecular variance (AMOVA) showed a low amount of variation between groups but a high amount within the populations analysed. Our analysis of molecular variance (AMOVA) showed a low amount of variation between groups, but a high amount within the analysed populations of hogweed in Georgia, Russia, Lithuania, and Poland. Our study confirms the results obtained by [53,54] for the three European invasive species (*H. sosnowskyi*, *H. mantegazzianum*, and *H. persicum*), where higher intrapopulation variability was found than in the native range. This phenomenon has previously been described as a result of hybridisation and inbreeding in the evolution of invasive species during their initial establishment and subsequent range expansion [24,55]. It is very likely that there were opportunities for intraspecific hybridisation after the introduction of Heracleum species, because they were introduced to Europe as garden ornamentals and a seed exchange was very popular in the 19th and early 20th centuries, not only between botanical gardens, but also between private gardeners [7]. In Poland and Lithuania, *H. sosnowskyi* has been cultivated extensively as a fodder species and seeds have been collected from various sources [11,56]. Similar patterns have been observed with other invasive species such as *Ambrosia artemisiifolia*, *Bunias orientalis* [57], and *Sisymbrium austriacum* subsp. *chrysanthum* [58].

### 4.2. Genetic Distance and Similarity

The smallest genetic distance was observed between *H. sosnowskyi* populations from Poland and Lithuania, which have an invasive status, and populations from Georgia and Lithuania. The Georgian population was located in the natural range of the species studied. This was probably the origin of the invasive population found in Lithuania. This was supported by the slightly larger statistically significant genetic distance between the Lithuanian and Russian populations (0.017, *p* = 0.048). The population of Sosnovsky’s hogweed in Poland was characterised by a larger, statistically significant genetic distance from the Russian population and a slightly smaller distance from the Georgian population. The smallest genetic distance occurs between natural Caucasian populations (Table 3). The positive correlation between geographical distance and genetic diversity of the analysed hogweed populations is confirmed by the high and statistically significant value of the Mantel coefficient (r = 0.716, *p* = 0.01).

The genetic similarity of the analysed hogweed populations is shown by a dendrogram representing the Dice similarity coefficient (Figure 4). The high bootstrap values indicate the high reliability of the analysis. Genetic differences are particularly evident in the Georgian populations, which are divided into two groups: the first (samples 54–58 form G1) shows high similarity to the Russian populations, the second (samples 49–53 from G2) is clearly distinct from the others originated from this geographical region and is genetically similar to the Lithuanian population, which is outside the natural range of the studied species and has an invasive status. The Polish population is clearly separated from the other populations studied and is highly differentiated internally. The results discussed above are confirmed by the PCoA, where origin is the grouping variable. Individual points represent single samples from a given location, and their density indicates genetic similarity. On this basis, three distinct populations were distinguished: a Russian one, a natural one correlated with axis 2 of the ordination diagram, and two invasive ones: a Lithuanian one negatively correlated with axis 1 and a Polish one positively correlated with the axis in question. The genotypes of the Georgian population show similarity with the Russian (samples 84 R2 and 90 R4) and Lithuanian (samples 59–67 L1–L3) populations (Figure 4).

### 4.3. Genetic Structure of Analysed Populations

Genetic structure analysis supports at least three allele pools within the analysed populations. Upon analysing the history of the spread of Sosnovsky’s hogweed, it was assumed that the Russian and Georgian populations from the Caucasus were natural [10,16,54]. The results of the Bayesian analysis showed that the Russian populations represent a single genetic lineage with minor admixtures from populations of the Georgian lineage (84 and 86–87 from R2 and 90 from R4). Georgian populations contain two genetic lineages. The Georgian population from Mccheta-Mtianetia–G2 [49,50,51,52,53] have their own individual character and differ from the Russian (R1–R4) lines. However, the population from Stepantsminda (G1) belongs to the Russian gene pool. This suggests a southward migration of hogweed and a founder effect, followed by the coexistence of the two genetic lines. This analysis also showed that the Lithuanian population belongs to the Georgian gene pool, is uniform in this respect, and is not related to the Polish gene pool. This suggests an independent introduction of Sosnovsky’s hogweed to Lithuania and Poland. The third gene pool was represented by hogweed genotypes from Poland. Some populations derived from different locations in the country (samples 1–4, 7–8, 10–12) have small admixtures of both Russian and Georgian lines, others possess exclusively Russian (samples 15, 16) or Georgian (samples 21, 32–33, 35–36) alleles. This suggests multiple migration episodes and a founder effect, but also the rapid evolution of hogweed in Poland, which has led to the isolation of a distinct genetic lineage.

There is a high probability that the native forms analysed did not include genotypes related to all populations that were used in the 1940s and 1950s in the breeding process to produce materials that were then distributed throughout the Soviet republics and other countries under the influence of the Soviet Union. The existing Georgian populations analysed are from the Caucasus region, where Mandenova [30] originally described it. Based on the obtained results, it seems that the Lithuanian populations come from a different genetic line or a different seed transfer than the Polish populations (Figure 5). The Lithuanian populations are related to one of the native populations from Georgia. The two Georgian populations differ from each other, probably because they come from different locations, separated from each other by the Caucasus mountain range, which is a significant geographical barrier. The Georgian population from Stepantsminda (G1) that clusters with the Russian populations is geographically closer to Russian populations than the population from Mccheta-Mtianetia (G2), which is more closely related to the Lithuanian (L1–L3) populations. The populations of *H. sosnowskyi* sampled in Europe analysed by Jahodová et al. [54] were distinct from those from the native distribution range.

*H. sosnowskyi* was introduced to Eastern Europe as a crop plant; seeds from the Republic of Kabardino-Balkaria (Russian Caucasus) and Dagestan were used in plant breeding programmes in NW Russia (Murmansk and Leningrad) [8]. The main goal of these programmes was to produce a high-yielding variety with a minimal content of furanocoumarins, a substance responsible for photodermatitis. Selection of these compounds may well have resulted in different European genotypes. Populations from Denmark, analysed by Jahodová et al. [10], clustered with those from Armenia, which suggests that this is an example of a separate introduction event, as could also be the case of the Lithuanian population.

### 4.4. Rapid Evolution and Adaptation

Many emerging invasive species display evidence of rapid adaptation. Humans typically facilitate the dispersal of the genus Heracleum, including *H. sosnowskyi*, but the final outcome of invasions can vary. Recent research on invasive species, e.g., [59], suggests that both ecological processes and contemporary evolution influence the invasion process, being either a success or a failure. For example, the composition of plant pathogens and herbivores differs between native and introduced regions. The absence of certain plant diseases and herbivores may partly explain the success of some invaders, as they can divert resources from defence to growth and reproduction. Allelopathy may be one of the mechanisms driving the invasion of some plants, including *H. mantegazzianum*, as proposed by Jandová et al. [60]. In addition, Higgins and Richardson [61] have shown that many invasive plants have broader physiological niches than other species. Another key factor in species adaptation is the time of introduction. Recent genetic studies demonstrate that adaptation to novel environments can occur within 20 generations or fewer, suggesting that evolutionary processes can influence invasiveness [3,4,62]. A study of *H. sosnowskyi* populations in the native and introduced range revealed considerable genetic variation within species despite a clear separation among populations and suggested multiple colonisation events as a plausible hypothesis explaining the contemporary history of the plant.

## 5. Conclusions

*H. sosnowskyi* have been used in agriculture since 1947 [30] as a fodder. Seeds of this species were derived from different native habitats, especially Central and Eastern Caucasus and Transcaucasia. These multiple origins and unrelated introductions resulted in a large number of founders. Following the introduction, rapid evolution, drift, or hybridisation are assumed to have played a role in the genetic structuring of these invading populations. The hogweed populations found in Poland are a good example of a potentially successful process of adaptation to local conditions and high potential to further spread, which may be associated with the observed genetic variability. However, conclusions regarding large-scale population structure should be interpreted with caution, as genetic differentiation between countries was low and only marginally non-significant. Caucasian hogweed’s invasiveness is increased by its ability to rapidly adapt to new habitats, and by its genetic diversity. ISSR markers allow for the assessment of genome-wide genetic variation, but at relatively low resolution. They are generally considered to reflect primarily neutral polymorphism. The variation detected in our study reflects general patterns of genetic differentiation but cannot be directly linked to functional genes underlying invasiveness.

## Figures and Tables

**Table 1:** The sampling sites with geographical and habitat characteristics of the population used in the study.

Population Name and Code	Coordinates	Area (ha)	Exhibition	Habitat	Date of Sampling	Altitude a.s.l.
GEORGIA (G) native populations						
Mccheta-Mtianetia G2	42°29′39.4″ N 44°27′11.3″ E	0.1	S	pasture	4 July 2023	1910
Stepantsminda G1	42°39′35.7″ N 44°38′25.6″ E	0.1	flatland	wasteland	5 July 2023	1820
RUSSIA (R) native populations						
Kislawodsk R4	43°54′47.4″ N 42°41′50.8″ E	0.04	S-W	forest	2 July 2023	800
Polyana Narzanov R2	43°14′45.3″ N 42°33′36.7″ E	0.06	S	forest	18 June 2023	1991
Tsey R3	42°47′07.8″ N 43°54′20.4″ E	0.03	N-E	grassland	23 June 2023	2393
Vodopad “Devichyji Kosy” R1	43°15′42.8″ N 42°30′25.5″ E	0.01	S	forest	20 June 2023	2496
LITHUANIA (L) secondary, invasive populations						
Nadaukali L3	55°49′26.1″ N 23°31′45.5″ E	0.3	flatland	wasteland	23 July 2023	117
Siauliai L1	56°00′53.9″ N 23°25′02.1″ E	0.01	flatland	meadow	21 July 2023	112
Taurogo L2	55°14′00.4″ N 22°13′12.1″ E	0.02	flatland	wasteland	18 July 2023	14
POLAND (P) invasive populations						
Gnojno P10	52°75′15.0″ N 23°14′84.6″ E	0.18	flatland	wasteland	19 August 2023	152
Grodkowice P5	49°58′34.1″ N 20°16′41.0″ E	0.1	S-E	meadow	25 June 2023	908
Kotorów P1	50°43′04.2″ N 23°43′55.3″ E	0.9	flatland	pasture	19 June 2023	277
Lublin P12	51°26′55.2″ N 22°51′58.7″ E	1.2	flatland	wasteland	16 August 2023	213
Machnowska Góra P9	50°36′37.5″ N 23°58′46.1″ E	0.6	flatland	wasteland	16 August 2023	235
Murowana Goślina P2	52°35′19.7″ N 17°00′51.5″ E	0.2	flatland	wasteland	24 August 2023	80
Ożenna P8	49°42′52.6″ N 21°45′65.1″ E	0.03	flatland	wasteland	19 August 2023	547
Powidz P4	52°24′13.7″N 17°54′40.6″E	0.01	flatland	wasteland	26 August 2023	96
Poznań P3	52°27′90.7″ N 16°57′47.4″ E	0.1	flatland	wasteland	28 July 2023	81
Ściborzyce P7	50°17′46.9″ N 19°53′56.9″ E	0.15	S	wasteland	26 June 2023	391
Trzyciąż P6	50°18′39.2″ N 19°45′59.9″ E	0.05	flatland	wet meadow	26 June 2023	355
Wisłok Wielki P11	49°41′26.0″ N. 21°97′98.8″ E	0.01	flatland	wasteland	19 August 2023	479

**Table 2:** List of ISSR primers of *H. sosnowskyi* DNA used in the study and values of markers polymorphism indices. Number of polymorphic fragments (NPF), polymorphism information content (PIC).

Primer	Sequence 5′→3′	NPF	PIC	Fragment Size Range (bp)
SR14	(GA)_7_YG	14	0.370	220–1250
SR17	(GA)_8_YC	12	0.293	280–1000
SR31	(AG)_8_YC	14	0.325	250–800
SR33	(AG)_8_T	15	0.365	250–1000
SR34	(TC)_8_CC	12	0.309	380–1060
SR35	(TC)_8_CG	7	0.378	450–1100
SR37	(AC)_8_C	11	0.341	420–770
SR40	(AC)_8_T	12	0.314	360–750
SR42	(AG)_8_YA	16	0.322	250–1200
SR45	(GA)_8_T	19	0.326	250–1300
SR46	(GA)_10_A	18	0.340	380–1180
SR49	(TC)_9_A	14	0.364	390–940
SR50	(TC)_9_C	14	0.355	400–1080
SR54	(CT)_9_T	12	0.433	550–1300
SR63	(ATG)_6_C	19	0.379	560–1900
SR64	(ATG)_6_AC	19	0.294	420–1750
SR65	(ATG)_6_T	16	0.385	350–1400
Mean		14	0.347	220–1250

**Table 3:** Intrapopulation genetic diversity of 21 *H. sosnowskyi* genotypes collected in Poland, Georgia, Lithuania and Russia using ISSR data.

Population	N	NB	%P	NPB	UHe
Georgia	10	186	66.39	0	0.235
Russia	20	198	74.18	1	0.224
Lithuania	18	193	62.70	4	0.209
Poland	48	210	77.05	6	0.254
Mean of all populations	197	70.08	2.75	0.231	

No. of specimens used in genetic analyses (N), number of bands (NB), percentage of polymorphic bands (%P), number of private markers present in population but absent in another population (NBP), unbiased expected heterozygosity (UHe).

**Table 4:** Summary of the molecular variance AMOVA results for 96 samples of *H. sosnowskyi.* (**a**) Genotypes grouped into populations by country of origin. (**b**) Genotypes grouped into native population (from Russia and Georgia) and invasive population (from Poland and Lithuania).

Source of Variation	df	SS	MS	Estimated Variance	%	*p* Value
(a)						
Among populations	3	1.799	0.600	0.005	1%	0.045
Within populations	92	44.889	0.488	0.488	99%	
(b)						
Among populations	1	0.847	0.847	0.009	2%	0.001
Among populations	1	0.847	0.847	0.009	2%	

df, degrees of freedom; SS, sum of squares; MS, mean sum of squares; *p* < 0.05.

**Table 5:** Pairwise population PhiPT genetic diversity values (below diagonal) and probability level based on 999 permutations (above diagonal) between *H. sosnowskyi*. Statistically significant differences *p* < 0.05. (**a**) Populations collected in different countries. (**b**) Native population (from Russia and Georgia) and invasive population (from Poland and Lithuania).

(a)				
**Population**	**Georgia**	**Russia**	**Lithuania**	**Poland**
Georgia		0.087	0.493	0.146
Russia	0.028		0.046	0.001
Lithuania	0.000	0.017		0.486
Poland	0.013	0.024	0.000	
(b)				
**Population**	**Native**		**Invasive**	
Poland and Lithuania			0.002	
Russia and Georgia	0.018			

## Data Availability

Data will be available from the corresponding author on reasonable request.

## Abbreviations

The following abbreviations are used in this manuscript:

DNA	Deoxyribonucleic Acid
PCR	Polymerase Chain Reaction
ISSR	Inter Simple Sequence Repeat
NPF	Number of Polymorphic Fragments
PIC	Polymorphism Information Content
He	Expected Heterozygosity
AMOVA	Analysis of Molecular Variance
PhiPT (ΦPT)	Genetic Differentiation Coefficient
UPGMA	Unweighted Pair Group Method with Arithmetic Mean
PCoA	Principal Coordinate Analysis
*H. sosnowskyi*	*Heracleum sosnowskyi*
